# Synthesis of novel chemicals from cardanol as a product of cashew nutshell processing

**DOI:** 10.1002/fsn3.1480

**Published:** 2020-02-25

**Authors:** Jens Deutsch, Angela Köckritz

**Affiliations:** ^1^ Leibniz Institute for Catalysis (LIKAT) Rostock Germany

**Keywords:** biomass conversion, heterogeneous catalysis, homogeneous catalysis, hydrogenation, oxidation

## Abstract

The conversion of the worldwide chemical production from fossil to sustainable resources is currently one of the most urgent tasks for the chemical industry. Based on this approach cardanol, a mixture of phenols with C15‐chains as substituents is produced in some countries of the tropical zone from the processing of cashew nutshells. The paper reports the specific transformation of the aromatic moiety in this cheap material, and thus, the development of a novel route to potential useful green bifunctional chemicals in gram scale. Accordingly, cardanol was converted successfully in three steps into hexane‐1,6‐diols. The evaluation of appropriate synthesis methods and suitable conditions for each of these reaction steps is presented as an essential topic of these investigations. The target compounds synthesized in the reaction sequence are potential building blocks for future biomass‐based chemicals and monomers for green polymeric materials, surfactants, and lubricants.

## Graphical abstract

The paper reports the specific chemical transformation of the aromatic moiety in cardanol as a product of cashew nutshell processing. Cardanol was converted successfully in three steps into hexane‐1,6‐diols. The final products synthesized in the reaction sequence are potential building blocks for novel biomass‐based chemicals and monomers for green polymeric materials, surfactants, and lubricants.

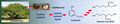

## 1 INTRODUCTION

Fats and oils isolated from the seeds or fruits of oil plants (e.g., sunflower, olive, rapeseed, flax) are important components of human nutrition. In addition, such materials are renewable starting materials for the sustainable production of novel chemicals. Unsaturated fatty acids are the base compounds of most fats and oils. They reveal a linear molecular structure with an unsaturated hydrocarbon chain and a terminal carboxyl group. Both structural units offer various possibilities for useful chemical transformations.

The refinement of unsaturated fatty acids and their derivatives (oleochemicals) is mainly focused on the commonly established chemistry of alkenes. Examples for such transformations are the epoxidation, dihydroxylation, oxidative cleavage, metathesis, hydroformylation, isomerizing hydroformylation, hydroaminomethylation, and isomerizing alkoxycarbonylation of the olefin moiety in the hydrocarbon chain (Seidensticker, Vorholt, & Behr, 2016). Recent contributions to this chemistry report the hydroalkylation (Biermann & Metzger, 2018) and the Wacker oxidation (von Czapiewski & Meier, 2018) of methyl oleate.

**Figure 1:**
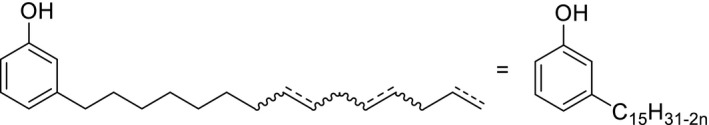
The components of cardanol (provider: Zhuhai Co. Ltd); *n* = 0 (2%), *n* = 1 (51%), *n* = 2 (14%), *n* = 3 (33%); average content of double bonds in the chain: 1.78; accordingly calculated molecular weight: 300.93 g/mol

In addition to the oil plants mentioned above, the cashew tree is another versatile agricultural crop. Besides edible cashew nuts as fruits, the remaining nutshells are a valuable precursor of “green” chemicals. Accordingly, the technical solvent extraction of ground cashew nutshells yields cashew nutshell liquid (CNSL) as intermediate. In 2013, about 1,000,000 tons of CNSL were produced (Parambath, 2017 ) mainly in the countries Brazil, India, Indonesia, Vietnam, and Thailand (Monisha, Amarnath, Mukherjee, & Lochab, 2019). The subsequently performed thermal treatment of CNSL provides a mixture of substituted phenols with the main component cardanol. Pure cardanol (CAS 37330‐39‐5) is isolated finally after vacuum distillation. This distillate consists of four substituted phenols, which contain a saturated (*n* = 0), unsaturated (*n* = 1), double unsaturated (*n* = 2), or triple unsaturated (*n* = 3) C15‐chain in metaposition to the hydroxyl group (Figure 1).

So far, plenty of chemicals have been synthesized from cardanol as starting material. By analogy with fatty acid chemistry, the alkene groups in the unsaturated C15 chain of cardanol were epoxidized to give useful chemicals. In addition, the phenolic moiety in the molecule was subjected to further chemical transformations, such as etherification and acylation of the hydroxyl group, and nitration and sulfonation of the aromatic ring (Caillol, 2018; Voirin et al., 2014). The generated compounds have been utilized as monomers, for example, cardanol benzoxazines as one example (Shukla, Mahata, Pathak, & Lochab, 2015). The variety of polymer materials like phenolic resins, epoxy resins, cyanate ester resins, benzoxazine resins, polyols, and polyurethanes obtained from cardanol‐based monomers is listed in three review articles (Monisha et al., 2019; Shukla et al., 2015; Voirin et al., 2014).

In contrast to these concepts, we have cleaved the aromatic ring of cardanol to synthesize hexane‐1,6‐diols as novel bio‐based bifunctional fine chemicals or monomers.

## 2 MATERIALS AND METHODS

### 2.1 Chemicals

The cardanol sample used in the experiments, Cardolite NX‐2026, was purchased by Evonik Creavis GmbH from Zhuhai Co. Ltd. The chemicals, solvents, and the heterogeneous catalysts 5%Ru/C, 5%Rh/C, 10%Pt/C, 5%Ru/Al_2_O_3_, 5%Rh/Al_2_O_3,_ and 10%Pd/Al_2_O_3_ were purchased from Sigma‐Aldrich. The heterogeneous catalysts 10%Pd/C and 5%Pt/Al_2_O_3_ were purchased from Acros Organics and Strem Chemicals, respectively. The homogeneous catalyst Ru‐MACHO‐BH was purchased from Strem Chemicals. All chemicals, solvents, and catalysts were used without further purification. Moistened heterogeneous catalysts were dried at 60°C before use.

### 2.2 Analytical methods

The NMR spectra were recorded on a Bruker AV 300 (300 MHz) or Bruker AV 400 (400 MHz) spectrometer in CDCl_3_. The chemical shifts reported are relative to the center of the solvent resonance. IR spectra were recorded on a Bruker Alpha FT‐IR spectrometer using the ATR technique. GC was performed on a Hewlett–Packard HP 6890 chromatograph (HP5 column, 30 m, temperature program 50‐8‐260/5‐8‐280/5‐8‐300/5). Mass spectra of products were determined on an Agilent 6890/5973 GC‐MS. Elemental analyses were performed using a Leco TruSpec Micro CHNS elemental analyzer.

### 2.3 Screening experiments and syntheses

Screening of heterogeneous catalysts in the hydrogenation of cardanol: Cardanol (1.505 g, 5 mmol), 15 ml cyclohexane, and the heterogeneous catalyst (with 0.089 mmol of the respective metal) were placed in a 100 ml stainless steel autoclave. Next, the autoclave was locked, pressurized three times with argon (20 bar), and subsequently three times with hydrogen (20 bar). The reaction mixture was then stirred (600 rpm) at room temperature for 24 hr. After careful expansion of the mixture, the obtained solution was filtered, the solvent was removed with a rotary evaporator, and the remaining material was analyzed with GC and GC‐MS.

Synthesis of 3‐pentadecylcyclohexan‐1‐one: Cardanol (4.514 g, 15 mmol), 12 ml cyclohexane, and the heterogeneous catalyst 10%Pd/C (with 0.024 g Pd, 0.225 mmol) were placed in a 100 ml steel autoclave. Next, the autoclave was locked, pressurized three times with argon (20 bar), two times with hydrogen (20 bar), and again with hydrogen (25 bar). The reaction mixture was then stirred (600 rpm) at room temperature for 2 hr, heated to 45°C, and hydrogenated at this temperature with continued stirring until the pressure had dropped to 7.5 bar (~24 hr). After cooling to room temperature and careful expansion of the mixture, the obtained solution was filtered and the solvent was removed with a rotary evaporator. The remaining colorless oil solidified slowly on standing. Subsequently, phenyl isocyanate (0.447 g, 3.75 mmol) was added to the gently molten material and the liquid mixture stored at room temperature for 12 hr. The final kugelrohr distillation (5 × 10^–3^ mbar, temperature of the heating tube: 205–220°C, done twice) gave 3.318 g of the target product 3‐pentadecylcyclohexan‐1‐one as white crystals (yield: 72%). ^1^H NMR: (300 MHz, CDCl_3_): *δ* (ppm) = 0.87 (t, *J* = 6.8 Hz, 3H), 1.20–1.39 (m, 29H), 1.55–1.83 (m, 2H), 1.84–2.11 (m, 3H), 2.17–2.47 (m, 3H); ^13^C NMR: (300 MHz, CDCl_3_): *δ* (ppm) = 14.1, 22.7, 25.3, 26.6, 29.3, 29.5, 29.6, 29.7, 31.3, 31.9, 36.6, 39.1, 41.5, 48.2, 212.2; IR (ATR): *ν* (cm^−1^) = 2,915, 2,849, 1,704; MS (EI) *m*/*z* (rel. int.): 308 (M^+·^, 1), 265 (2), 97 (100), 83 (4), 69 (6), 55 (13), 41 (8); Elemental analysis: C_21_H_40_O (308.55): calcd. C 81.75, H 13.07; found C 81.68, H 13.33; GC purity: 97.4 area %.

Screening experiments on the oxidation of 3‐pentadecylcyclohexan‐1‐one with formic acid/ hydrogen peroxide: 3‐Pentadecylcyclohexan‐1‐one (1.543 g, 5 mmol) was dissolved in a stirred mixture of 6 ml 1,2‐dichloroethane and 95% formic acid (0.485 g, 10 mmol; 0.727 g, 15 mmol; or 0.969 g, 20 mmol, respectively). A solution of 50% hydrogen peroxide (0.680 g, 10 mmol; 1.021 g, 15 mmol; 1.362 g, 20 mmol; or 2.723 g, 40 mmol, respectively) was added in three portions within 15 min. Subsequently, the stirring was continued at room temperature for 24 hr (thin‐layer chromatography: complete conversion of the starting compound), and the solution was diluted with 15 ml of 1.2‐dichloroethane, filtered and extracted with 10 ml water (done twice). After the addition of the GC‐standard n‐tetradecane (0.496 g, 2.5 mmol), the solution was dried with anhydrous sodium sulfate, filtered and analyzed with GC.

Synthesis of 6‐pentadecyl‐ε‐caprolactone and 4‐pentadecyl‐ε‐caprolactone (as isomer mixture): 3‐Pentadecylcyclohexan‐1‐one (1.543 g, 5 mmol) was dissolved in 5 ml of 1,2‐dichloroethane, and m‐chloroperbenzoic acid (1.253 g, 6 mmol; moistened, 75%) was added to the solution in five portions during 90 min. The mixture was stirred at room temperature for 16 hr and afterward at 50°C for 2 hr. Subsequently, the cooled solution was filtered to separate the precipitated by‐product m‐chlorobenzoic acid and the solvent was removed in vacuo (<50°C). The obtained residue was dissolved in 20 ml of toluene, the solution was extracted with 15 ml 5% sodium bisulfite (done twice), and the turbid organic phase was filtered again. The clear filtrate was washed with 15 ml of 10% sodium hydrogen carbonate (done twice) and 15 ml of water. Finally, the organic solution was dried with anhydrous sodium sulfate, filtered and the solvent was removed in vacuo to give 1.431 g pure 6‐pentadecyl‐ε‐caprolactone and 4‐pentadecyl‐ε‐caprolactone as tan crystal mass (yield: 88%). ^1^H NMR: (400 MHz, CDCl_3_): *δ* (ppm) = 0.88 (t, *J* = 6.9 Hz, 3H), 1.08–1.52 (m, 29H), 1.59–1.83 (m, 2H), 1.83–2.02 (m, 2H), 2.48–2.71 (m, 2H), 3.98–4.30 (m, 2H); ^13^C NMR: (400 MHz, CDCl_3_): *δ* (ppm) = 14.1, 21.4, 22.7, 26.8, 26.9, 27.7, 29.4, 29.5, 29.6, 29.7, 31.6, 31.9, 34.0, 34.4, 34.9, 35.0, 36.0, 38.6, 40.1, 69.2, 72.8, 175.3, 176.1; IR (ATR): *ν* (cm^−1^) = 2,916, 2,849, 1,720; MS (EI) *m*/*z* (rel. int.): 324 (M^+·^, 0.3), 113 (100), 95 (6), 83 (12), 69 (8), 55 (14), 43 (12); 324 (M^+·^, 2), 306 (5), 128 (19), 111 (13), 97 (34), 88 (100), 69 (29), 55 (39), 43 (29); Elemental analysis: C_21_H_40_O_2_ (324.55): calcd. C 77.72, H 12.42; found C 77.88, H 12.37; GC purity: 95.8 area %.

Synthesis of 2‐pentadecylhexane‐1,6‐diol and 3‐pentadecylhexane‐1,6‐diol (as isomer mixture): 6‐pentadecyl‐ε‐caprolactone, 4‐pentadecyl‐ε‐caprolactone (1.623 g, 5 mmol), and Ru‐MACHO‐BH (0.029 g, 0.05 mmol) were dissolved under argon in 15 ml of diethylene glycol dimethyl ether. Next, the autoclave was locked and pressurized three times with argon (20 bar), two times with hydrogen (20 bar), and finally again with hydrogen (50 bar). The reaction mixture was then stirred (600 rpm) for 24 hr at 180°C. After cooling to room temperature and careful expansion of the mixture, the obtained solution was filtered through CELITE, the solvent was removed with a rotary evaporator, and the remaining material was purified by flash chromatography (Kieselgel 60, eluent: n‐heptane/ethyl acetate/ethanol mixture 6:4:1) to give 1.596 g 2‐pentadecylhexane‐1,6‐diol and 3‐pentadecylhexane‐1,6‐diol as white waxy solid (yield: 97%). ^1^H NMR: (300 MHz, CDCl_3_): *δ* (ppm) = 0.88 (t, *J* = 6.7 Hz, 3H), 1.18–1.41 (m, 31H), 1.42–1.65 (m, 4H), 2.14 (s, br, 2H), 3.44–3.73 (m, 4H); ^13^C NMR: (300 MHz, CDCl_3_): *δ* (ppm) = 14.1, 22.7, 22.8, 26.6, 27.0, 29.3, 29.5, 29.6, 29.7, 30.1, 30.5, 31.0, 31.9, 32.9, 33.8, 34.0, 36.7, 40.4, 60.9, 62.6, 63.1, 65.3; IR (ATR): *ν* (cm^−1^) = 3,313, 2,917, 2,849; MS (EI) *m*/*z* (rel. int.): 328 (M^+·^, 0.03), 280 (19), 125 (18), 111 (37), 97 (75), 83 (87), 69 (87) 55 (100), 43 (77); Elemental analysis: C_21_H_44_O_2_ (328.58): calcd. C 76.76, H 13.50; found C 76.67, H 13.67; GC purity: >99 area %.

## 3 RESULTS AND DISCUSSION

### 3.1 Selective hydrogenation of cardanol to 3‐pentadecylcyclohexan‐1‐one

**Figure 2:**
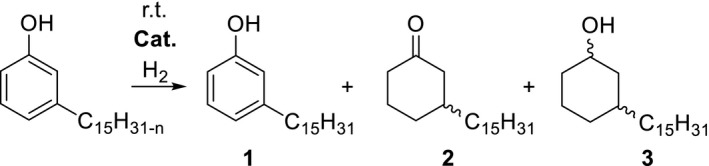
Screening of heterogeneous catalysts for the selective hydrogenation of cardanol

The first step within the scope of our synthesis strategy was the conversion of cardanol into the resulting cyclohexanone. According to the literature, a two‐step procedure was developed to generate the ketone 3‐pentadecylcyclohexan‐1‐one (Shingte & Wadgaonkar, 2004). Hence, cardanol was hydrogenated completely to 3‐pentadecylcyclohexan‐1‐ol followed by its oxidation to the desired ketone. It is noteworthy to mention in this context that the successful oxidation demanded the stoichiometric application of highly toxic chromium compounds. In front of this background, the direct selective hydrogenation of cardanol to 3‐pentadecylcyclohexan‐1‐one was identified as an environmentally friendly alternative and as an attractive task for our preliminary investigations (Figure 2). Thus, we tested various heterogeneous noble metal catalysts in this target reaction (Table 1).

**Table 1:** Screening of heterogeneous catalysts for the selective hydrogenation of cardanol

Entry	Catalyst^a^	Yields of the formed reaction products		
		**1** [area% GC]	**2** [area% GC]	**3** [area% GC]
1	5%Ru/Al_2_O_3_	30	0	0
2	5%Rh/Al_2_O_3_	0	0	>99
3	10%Pd/Al_2_O_3_	96	2	0
4	5%Pt/Al_2_O_3_	36	0	62
5	5%Ru/C	93	0	0
6	5%Rh/C	0	0	94
7	10%Pd/C	64	30	4
8	10%Pt/C	0	0	88

a Containing 1.78 mol% of the respective metal in relation to cardanol.

Despite the mild reaction temperature, all catalysts were found to be active in the hydrogenation of cardanol and gave, depending on the metal component, differently composed product mixtures (Table 1). The hydrogenation products 3‐pentadecylphenol **1** (entries 3, 5) and 3‐pentadecylcyclohexan‐1‐ol **3** (as diastereomer mixture, entries 2, 6, 8) were synthesized selectively with excellent yields. As an exception, the catalyst 10%Pd/C was able to produce the target product 3‐pentadecylcyclohexan‐1‐one **2** in a relevant amount (entry 7).

**Figure 3:**
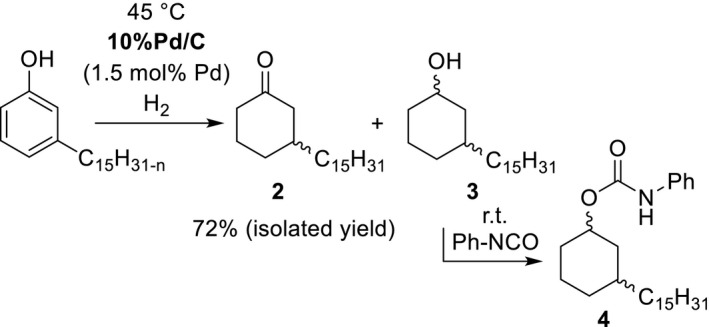
Selective heterogeneously catalyzed hydrogenation of cardanol to 3‐pentadecylcyclohexan‐1‐one **2** with subsequent conversion of the side product **3** into the separable compound **4**

With respect to the experimental results reported in Table 1, the hydrogenation of cardanol was repeated over 10%Pd/C using a moderately increased reaction temperature (Figure 3) to promote the formation of **2**.

We were able to show that the ketone **2** is available in good yield by hydrogenating cardanol at 45°C with careful control of the hydrogen consumption. Nevertheless, the obtained crude product contained some of the consecutively formed alcohol **3**. Its formation was found to be unavoidable and could not even be excluded for the hydrogenation experiment at room temperature (entry 7 of Table 1). To achieve an effective and quick separation of **2** from **3**, the latter was converted with an equimolar amount of phenyl isocyanate into the corresponding phenyl urethane **4** (Figure 3). The subsequent short path distillation of the mixture gave the desired ketone **2** in high purity. For the direct separation of **2** from **3** in technical scale, a fractionated distillation would clearly be cheaper and more sustainable. Experiments at temperatures above 50°C to accelerate the hydrogenation resulted contradictory to recent investigations (Rahobinirina et al., 2017) in an increased formation of **3** at the expense of **2**. In our experience, the selective hydrogenation of cardanol to **2** is highly temperature‐dependent. The identification of 10%Pd/C as appropriate selective hydrogenation catalyst is in accordance with various publications which report hydrogenations of the unsubstituted cardanol analogue phenol to cyclohexanone over heterogeneous Pd catalysts (Cheng, Dai, Li, & Wang, 2014; Feng, Liu, Chen, & Lou, 2014; Nelson, Manzano, Sadow, Overbury, & Slowing, 2015; Perez, Fajardo, & Corma, 2011; Zhou et al., 2017).

### 3.2 Oxidation of 3‐pentadecylcyclohexan‐1‐one to 5‐pentadecyl‐ε‐caprolactone and 3‐pentadecyl‐ε‐caprolactone

**Figure 4:**
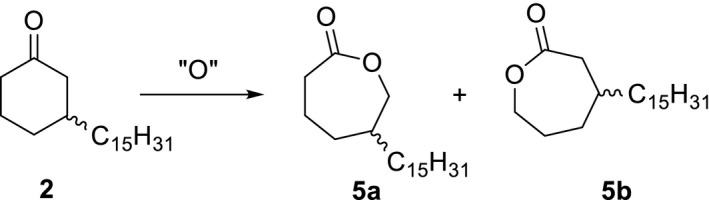
Oxidation of **2** to 5‐pentadecyl‐ε‐caprolactone **5a** and 3‐pentadecyl‐ε‐caprolactone **5b** [Correction added on 10 March 2020, after first online publication: Figure 4 has been updated.]

Subsequent to the selective hydrogenation of cardanol, the ketone **2** was to be oxidized to the related lactone in a second reaction step. With respect to the unsymmetrical molecular structure of **2**, the reaction was expected to give a mixture of two lactone isomers 5‐pentadecyl‐ε‐caprolactone **5a** and 3‐pentadecyl‐ε‐caprolactone **5b** (Figure 4).

In a relevant context, Pande and Gupta reported the successful oxidation of the parent compound cyclohexanone to ε‐caprolactone using a mixture of sodium tetraborate and 30% hydrogen peroxide as oxidizing agent (Pande & Gupta, 1995). The reaction was performed at 55°C in the solvent system 1,2‐dichloroethane/ water in presence of a phase transfer catalyst and gave the lactone in quantitative yield. Regrettably, we did not observe any conversion of **2** under these conditions.

The promising publication of Berkessel and Andreae describes the synthesis of ε‐caprolactone from cyclohexanone with excellent yield by means of 50% hydrogen peroxide in presence of various acids as catalysts at 60°C (Berkessel & Andreae, 2001). Remarkably, the use of the solvent 1,1,1,3,3,3‐hexafluoro‐2‐propanol (HFIP) was found to be essential for the oxidation. More detailed investigations revealed the formation of a spiro‐bisperoxide with a characteristic ^13^C NMR signal at 110 ppm from the ketone and the oxidizing agent before the acid was added. The authors concluded that the reaction mechanism of the oxidation is different from that of the Baeyer–Villiger reaction (Berkessel, Andreae, Schmickler, & Lex, 2002a, 2002b). In contrast to these papers, we did not observe any chemical reaction when hydrogen peroxide was given to **2** in HFIP. The subsequent addition of the catalyst p‐toluenesulfonic acid resulted in the precipitation of a solid. The isolated material displayed a ^13^C NMR signal at 109 ppm which might be assigned to the spiro‐bisperoxide derived from **2**. The continued stirring of the reaction mixture at 60°C yielded a gradual dissolution of this compound with formation of numerous undefined decomposition products instead of **5a** and **5b**. Eventually, our efforts to utilize Berkessel's procedure for the selective oxidation of **2** were not successful.

Molecular oxygen is a natural constituent of the atmosphere and in so far a very cheap chemical. Because of its poor reactivity for ketone oxidations, the gas is used in combination with sacrificial aldehydes to generate peracids as actual and more effective oxidizing agents. In this manner, 2‐ and 4‐methylcyclohexanone were oxidized to the resulting ε‐caprolactones at room temperature in 1,2‐dichloroethane as solvent with oxygen (1 atm) in presence of benzaldehyde (3 eq.) and copper(II) acetate (1 mol%) as catalyst (Bolm, Schlinghoff, & Weickhardt, 1993). Mastrorilli and Nobile demonstrated the usefulness of isobutyraldehyde as sacrificial aldehyde and of iron(III) 2‐(acetoacetoxy)ethyl methylacrylate as catalyst for the conversion of cyclohexanone into ε‐caprolactone under analogue conditions (Mastrorilli & Nobile, 1994). Inspired by these publications, we planned to transfer the above method to the oxidation of **2** to **5a** and **5b**. Our evaluation of copper(II) acetate and copper(II) acetylacetonate (1 mol%, respectively) as potential oxidation catalysts in combination with benzaldehyde as sacrificial reagent revealed conversion degrees above 90% for **2** as starting compound. It should be added that a reference experiment in absence of the metal salt gave the same result. The GC‐MS analyses of the reaction samples showed distinct signals for the target compounds **5a** and **5b,** but their apparent formation was not confirmed by corresponding NMR spectra. We assume that the detected lactones **5a** and **5b** were formed exclusively under the pyrolytic conditions in the GC injector from an unidentified thermally labile initial oxidation product. For that reason and in agreement with our NMR results, the desired lactones **5a** and **5b** are obviously not accessible from the ketone **2** and oxygen. Additional experiments using iron(II) acetate, iron(III) acetylacetonate, i‐butyraldehyde, and n‐hexylaldehyde for the target reaction did not show any improvement.

Peroxy acids (peracids) are known as efficient oxidizing agents of high reactivity. Baeyer and Villiger reported in their pioneering publication the utilization of peroxysulfuric acid (Caro's acid) for the oxidation of menthone, tetrahydrocarvone, and cyclohexanone to the corresponding ε‐caprolactones (Baeyer & Villiger, 1899). In the course of time, various organic peracids have, inter alia, been included successfully into this methodology (Renz & Meunier, 1999; ten Brink, Arends, & Sheldon, 2004). As an example, performic acid is generated in situ when hydrogen peroxide is added to a solution of formic acid and may be handled safely in this way. Performic acid was applied for the oxidation of 2‐ethoxyethyl cyclohexanone (Paust et al., 1993). Because of using formic acid in large excess (as solvent) for the mentioned procedure, the sensitive ester bond of the lactone was cleaved. To avoid this, our experiments with **2** were carried out in 1,2‐dichloroethane as solvent with reduced excess of formic acid (Table 2). The used halogenated solvent is resistant to the strong oxidant and can be recycled.

**Table 2:** Oxidation of **2** with peracids to a mixture of 5‐pentadecyl‐ε‐caprolactone **5a** and 3‐pentadecyl‐ε‐caprolactone **5b**

Entry	Peracid	Equivalents of the oxidizing agent		Yield^a^ of **5a** + **5b** b [%]
1	Performic acid	4 (HCOOH)	2 (H_2_O_2_)	58
2	Performic acid	3 (HCOOH)	3 (H_2_O_2_)	66
3	Performic acid	2 (HCOOH)	4 (H_2_O_2_)	72
4	Performic acid	2 (HCOOH)	8 (H_2_O_2_)	76
5	m‐chloroperbenzoic acid	1.2		88 (isolated yield)

a Yield according to GC analysis.

b Ratio 61:39 or vice versa.

According to entries 1–4, the ketone **2** was oxidized successfully with performic acid to the lactones **5a** and **5b** in moderate and good yields. In contrast, the oxidation with peracetic acid was reported to be less effective because of a comparatively poor yield of 39% (Rahobinirina et al., 2017). For all experiments compiled in Table 2, a complete conversion of **2** was observed. Thus, the ^13^C NMR spectra of the raw product mixtures exhibited distinct signals at 175 and 176 ppm for the carbonyl carbon of **5a** and **5b** and another signal at 112 ppm which may be assigned to a peroxide of unknown structure as a side product of the oxidation. Varying experiments with increasing excess of hydrogen peroxide in the oxidizing agent showed a positive impact on the target product yield (Table 2, entries 3 and 4).

Various groups reported the oxidation of various cyclohexanones with m‐chloroperbenzoic acid to ε‐caprolactones (Keck, Dougherty, & Savin, 1995; Romney, Colvin, & Miller, 2014). This oxidizing agent proved to be the best of all which have been tested in our experiments. In consequence, the ketone **2** was transferred completely into **5a** and **5b** in very good yield (Table 2, entry 5) and high analytical purity without any side products. Our efforts to separate the single isomers by means of flash chromatography were not successful and resulted in the decomposition of the lactones **5a** and **5b** to give a complex mixture of different compounds.

Besides cardanol, unsaturated fatty acids are also convertible into lactones. For oleic acid, an acid‐catalyzed intramolecular reaction is applied, initiated by the isomerization of the internal double bond, and completed by the cyclization of the intermediate to 4‐tetradecyl‐γ‐butyrolactone as thermodynamically favored compound (Cernak & Isbell, 2000; Gooßen, Ohlmann, & Dierker, 2010; Zhou, Keith, & Angelici, 2007). In contrast, the intramolecular lactonization of ricinoleic acid is performed by the transformation of its hydroxyl group on C12 into an ester group to give a macrocyclic target product (Nagarajan, 1999; Slivniak & Domb, 2005).

### 3.3 Hydrogenation of 5‐pentadecyl‐ε‐caprolactone and 3‐pentadecyl‐ε‐caprolactone to 2‐pentadecylhexane‐1,6‐diol and 3‐pentadecylhexane‐1,6‐diol

**Figure 5:**
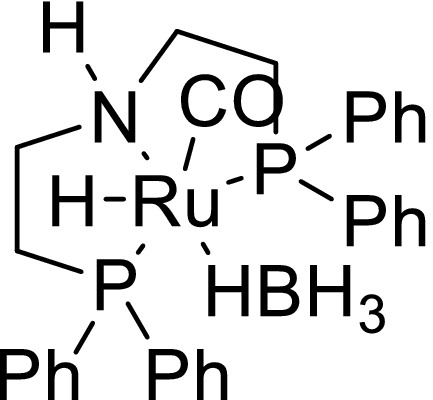
The hydrogenation catalyst Ru‐MACHO‐BH [Correction added on 10 March 2020, after first online publication: Comment balloon has been deleted in Figure 5.]

Finally, we planned the hydrogenation of **5a** and **5b** to their corresponding hexane‐1,6‐diols. The state of the art for ester hydrogenations had recently been summarized in two review articles (Pritchard, Filonenko, Putten, Hensen, & Pidko, 2015; Werkmeister, Junge, & Beller, 2014). In respect of our task, we identified the catalytic hydrogenation of ε‐caprolactone, the parent compound of **5a** and **5b**, as an instructive starting point for our experiments. Up to now, this reaction was found to be feasible at 150°C and 100°C in presence of [Mo(CO)_6_]/ [Rh(acac)(CO)_2_] and [RuCl_2_(PPh_3_)HN(C_2_H_4_SEt)_2_], respectively (Behr & Brehme, 2002; Spasyuk, Smith, & Gusev, 2013). We performed the hydrogenation of ε‐caprolactone using the buyable catalyst Ru‐MACHO‐BH (Kuriyama, Matsumoto, Ino, & Ogata, 2011; Figure 5). The expected product hexane‐1.6‐diol was generated at 70°C (50 bar hydrogen, 24 hr, catalyst loading: 1 mol%) in quantitative yield.

**Figure 6:**
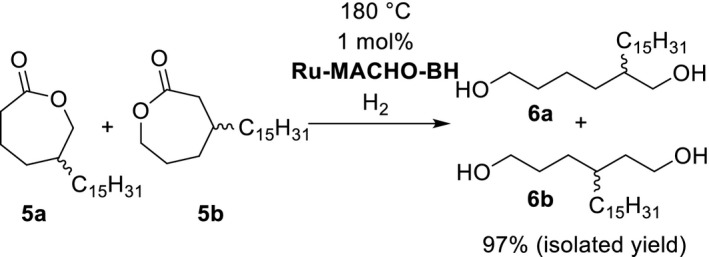
Homogeneously catalyzed hydrogenation of 5‐pentadecyl‐ε‐caprolactone **5a** and 3‐pentadecyl‐ε‐caprolactone **5b** to 2‐pentadecylhexane‐1,6‐diol **6a** and 3‐pentadecylhexane‐1,6‐diol **6b**

Fortunately, the same method was applicable for the desired conversion of **5a** and **5b** into the hexane‐1,6‐diols **6a** and **6b** (Figure 6).

The formerly identified high thermal stability of Ru‐MACHO‐BH (Deutsch, Pinto, Doerfelt, Martin, & Köckritz, 2015) revealed as beneficial catalyst quality since the hydrogenation of **5a** and **5b** demanded the considerably high reaction temperature of 180°C (Figure 6). We assume that the long C15‐chains of the cardanol derived lactones are probably responsible for their dramatically decreased reactivity. A further advantage of the catalyst Ru‐MACHO‐BH was the redundancy of usually required basic additives, such as alkali alkanoates, for Ru‐catalyzed ester hydrogenations.

## 4 CONCLUSIONS

In summary, we converted the biomass‐based oil cardanol in three reaction steps [(a) heterogeneously catalyzed hydrogenation, (b) oxidation, (c) homogeneously catalyzed hydrogenation] into a binary mixture of substituted hexane‐1,6‐diols as potential green building blocks or monomers. In the first step, the temperature‐controlled selective hydrogenation of cardanol to the resulting cyclohexanone derivative was developed as an environmentally friendly alternative to previously known laborious synthesis procedures. In the second and third step of our reaction sequence, we observed a comparatively poor reactivity for the cardanol derived starting compounds with C15‐chains. The identification of appropriate and correspondingly more drastic conditions for these reactions turned out to be the essential challenge of the presented work. In view of our investigations, the transformation of biomass‐based compounds with complex molecular structure into novel useful chemicals will be more demanding than that of their mineral oil‐based parent compounds. This is an ambitious but nevertheless an indispensable task for future chemistry.

## CONFLICT OF INTEREST

The authors declare that they have no conflict of interest.

## ETHICAL APPROVAL

This study does not involve any human or animal testing.
